# Supplementary figures and images for: The H2S–Nrf2–Antioxidant Proteins Axis Protects Renal Tubular Epithelial Cells of the Native Hibernator Syrian Hamster from Reoxygenation-Induced Cell Death
Source: Biology (Basel). 2019 Sep 30;8(4):74. doi: 10.3390/biology8040074 (PMC6955957; doi:10.3390/biology8040074)

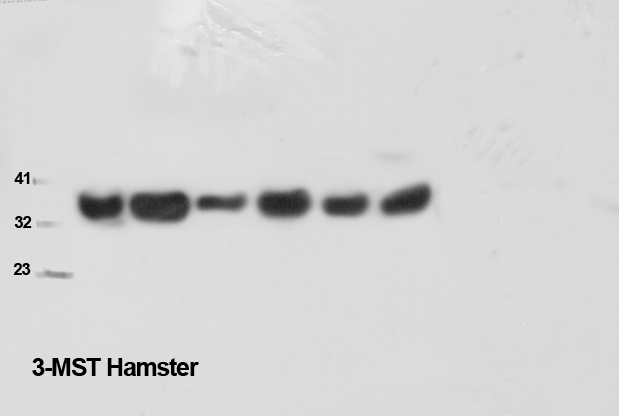

Supplement: Supplementary file 1 [file biology-08-00074-s001.zip › Scanned for Supplemetal/3-MST Hamster Scanned.tif]

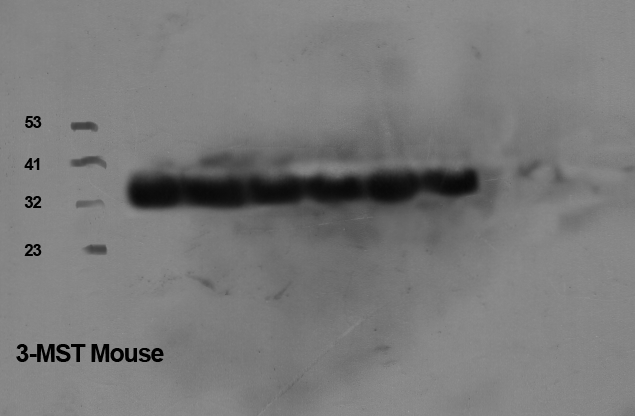

Supplement: Supplementary file 1 [file biology-08-00074-s001.zip › Scanned for Supplemetal/3-MST Mouse Scanned.tif]

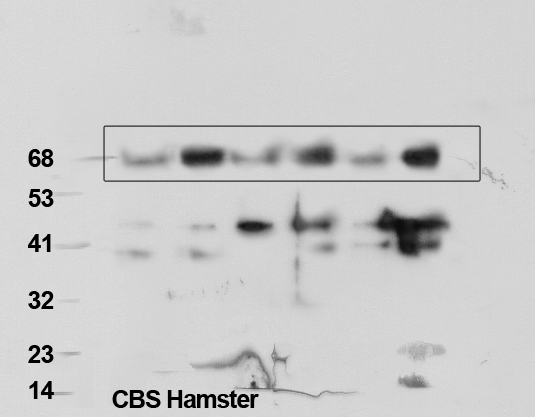

Supplement: Supplementary file 1 [file biology-08-00074-s001.zip › Scanned for Supplemetal/CBS Hamster Scanned.tif]

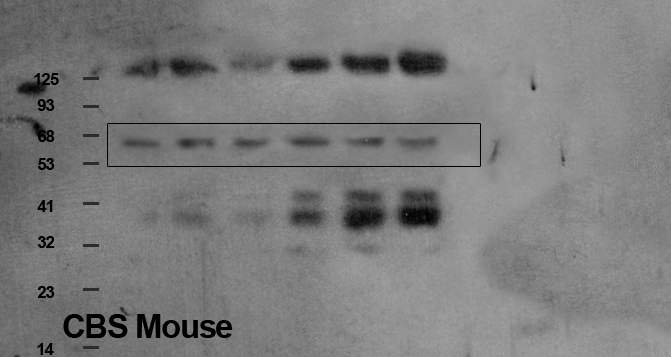

Supplement: Supplementary file 1 [file biology-08-00074-s001.zip › Scanned for Supplemetal/CBS Mouse Scanned.tif]

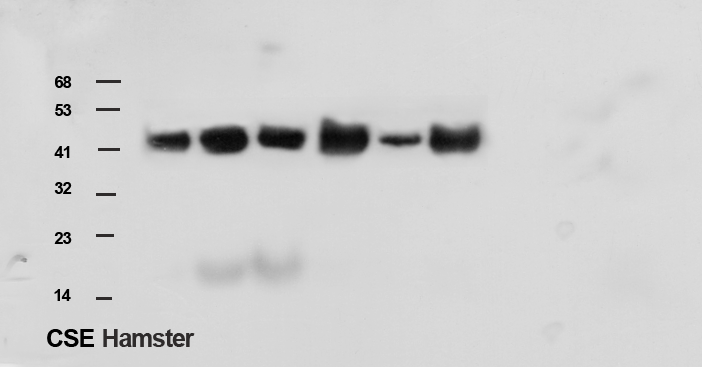

Supplement: Supplementary file 1 [file biology-08-00074-s001.zip › Scanned for Supplemetal/CSE Hamster Scanned.tif]

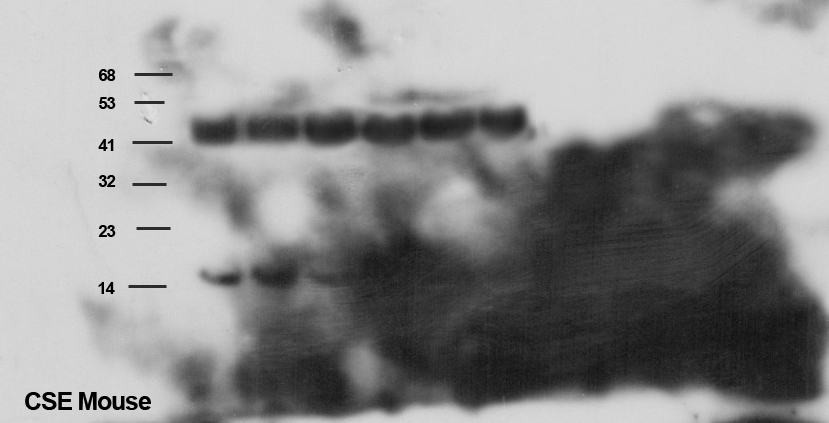

Supplement: Supplementary file 1 [file biology-08-00074-s001.zip › Scanned for Supplemetal/CSE Mouse Scanned.tif]

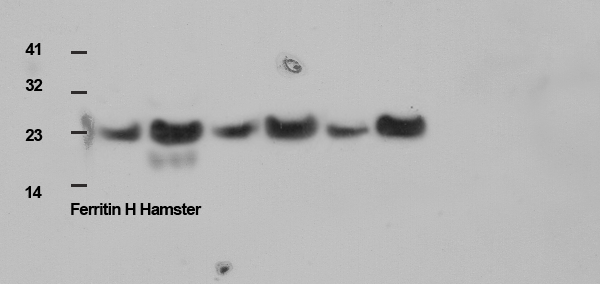

Supplement: Supplementary file 1 [file biology-08-00074-s001.zip › Scanned for Supplemetal/Ferritin Hamster Scanned.tif]

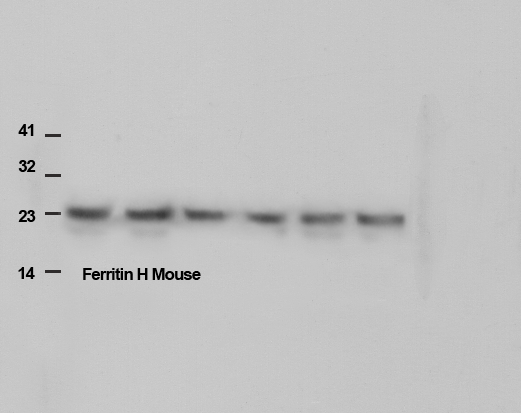

Supplement: Supplementary file 1 [file biology-08-00074-s001.zip › Scanned for Supplemetal/Ferritin Mouse Scanned.tif]

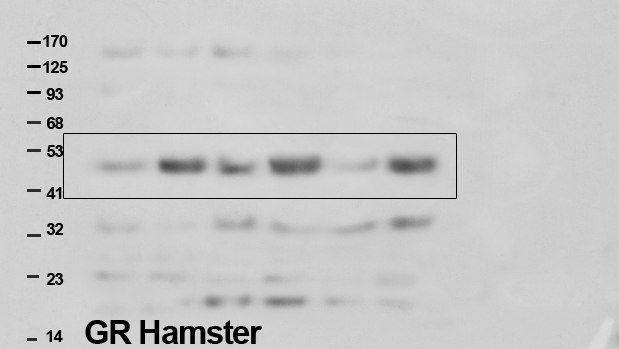

Supplement: Supplementary file 1 [file biology-08-00074-s001.zip › Scanned for Supplemetal/GR Hamster Scanned.tif]

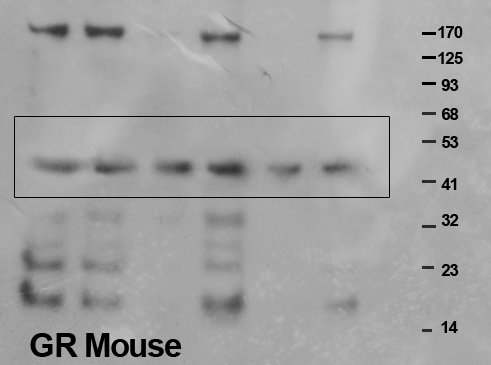

Supplement: Supplementary file 1 [file biology-08-00074-s001.zip › Scanned for Supplemetal/GR Mouse Scanned.tif]

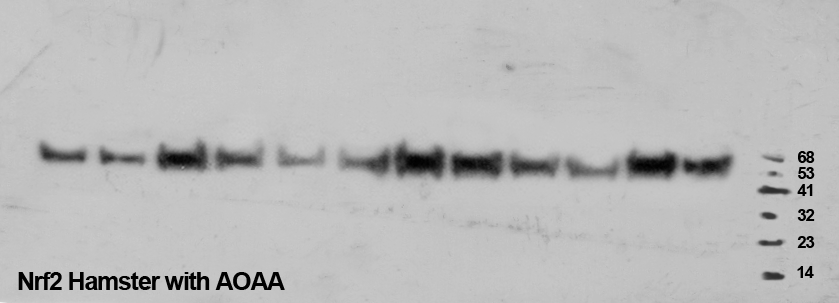

Supplement: Supplementary file 1 [file biology-08-00074-s001.zip › Scanned for Supplemetal/Nrf2 Hamster AOAA Scanned.tif]

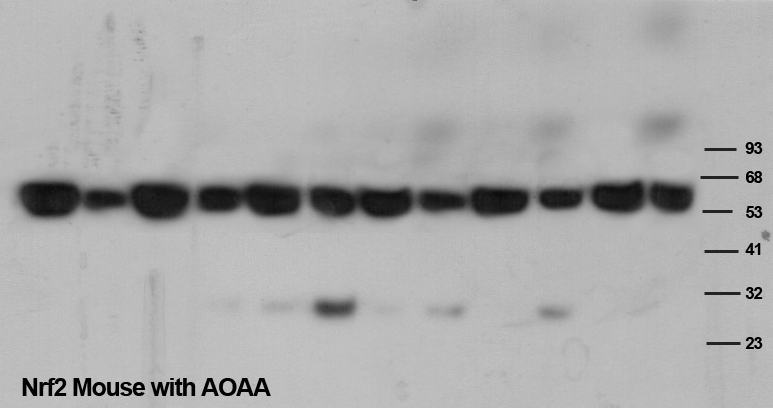

Supplement: Supplementary file 1 [file biology-08-00074-s001.zip › Scanned for Supplemetal/Nrf2 Mouse with AOAA Mouse Scanned.tif]

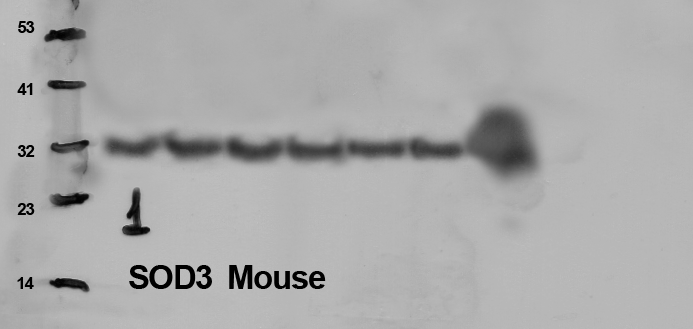

Supplement: Supplementary file 1 [file biology-08-00074-s001.zip › Scanned for Supplemetal/SOD 3 Mouse Scanned.tif]

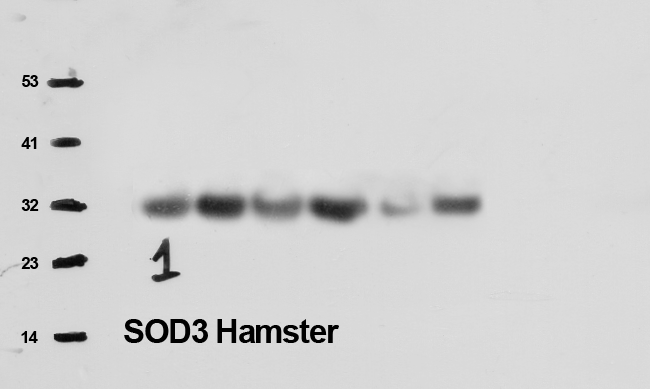

Supplement: Supplementary file 1 [file biology-08-00074-s001.zip › Scanned for Supplemetal/SOD Hamster Scanned.tif]

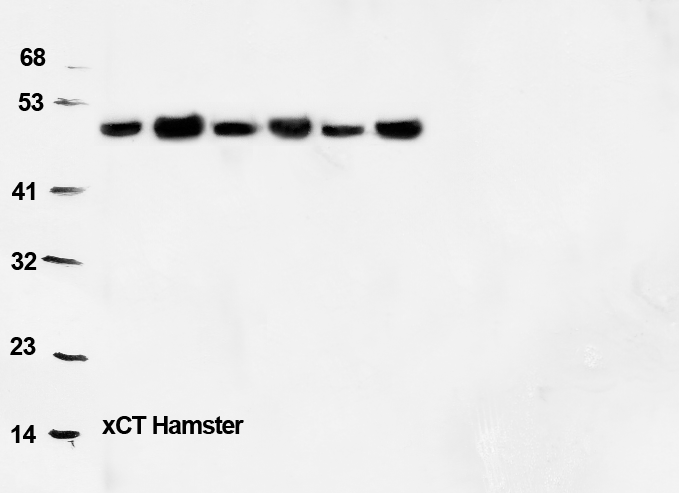

Supplement: Supplementary file 1 [file biology-08-00074-s001.zip › Scanned for Supplemetal/xCT Hamster Scanned.tif]

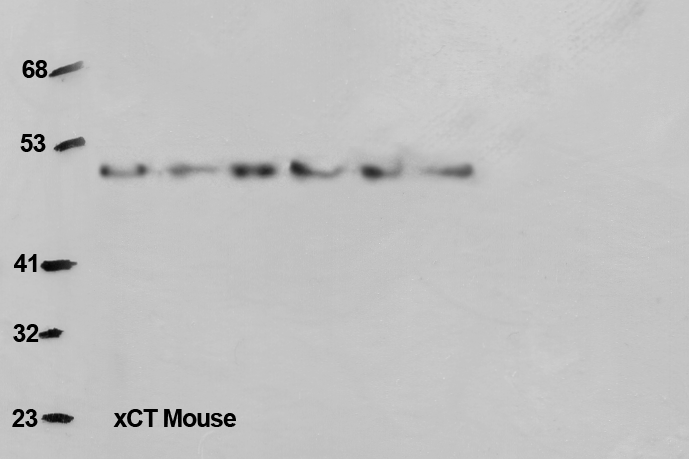

Supplement: Supplementary file 1 [file biology-08-00074-s001.zip › Scanned for Supplemetal/xCT Mouse Scanned.tif]
